# Gynæcological Medicine

**Published:** 1871-06

**Authors:** T. S. Powell, W. T. Goldsmith


THE GEORGIA

### MEDICAL COMPANION:

A MONTHLY ADVISER.

VOL. I. ATLANTA, GA., JUNE, 1871. No. 6.

### PART I.

### Original Communications and Special Selections.

### GYNÆCOLOGICAL MEDICINE.

*Report of the Committee of the Seventh Congressional District of Georgia, upon "Gynæcological Medicine," prepared to be read before the Georgia Medical Association. By T. S. Powell, M. D., and W. T. Goldsmith, M. D.*

[CONTINUED FROM LAST NUMBER.]

The treatment for ulceration of the os uteri may be divided under two heads—general and local. The first consisting of remedies and hygienic measures for restoration of constitutional vigor. The second of local applications and medicaments to the diseased part. Under the first division, tonics, alteratives, anodynes, etc., are appropriate. The impoverishment of blood is usually great, requiring ferruginous preparations, combined with alteratives, as iodine, etc. We have used arsenic with great advantage, and we regard aconite as a valuable remedy, in many cases. Dr. Merrell, of New York, highly recommends bebereena, attributing to it almost specific power over the diseases of the os uteri. Bromide of potassium and hydrate of chloral, in many cases, will be found of great value in controlling nervous excitement and hysterical phenomena. Rest, as a general remedy, is all-important—rest from manual and other labor, and rest of the parts from the mechanical irritation and excitement of sexual intercourse. Good, nutritious food, with fresh air and gentle exercise should be insisted upon as very important factors in the treatment. Under the head of chronic cervical endometritis we will notice more particularly the general treatment to be employed—ulcerations of the vaginal cervical mucus membrane being but forms of cervical endometritis.

The local treatment will depend, however, to the extent of limitation of the ulceration to the vaginal cervix—being most generally complicated with either chronic cervical endometritis, or corporeal endometritis. The local application of caustics will be similar in either form of cervical endometritis—either of the os or the canal—the method of application alone being changed, the former usually of easy approach, the latter requiring often dilitation of the os and canal, and instruments specially adapted in order to facilitate their application.

The question as to the frequency with which caustics should be applied, is one of great practical importance upon which depends, in a great degree, our success in the treatment of these diseases. According to our experience, no remedial measures employed by the gynæcologist, require more skill, tact, or whatever it may be called, than the application of alterative remedies for the purpose of changing the nutrition and overcoming the morbid condition of the cervix uteri. To cauterize the parts is easy—indeed this is always done by the inexperienced—but to give exactly the necessary stimulation ; to change the diseased parts from a morbid to a healthy condition—is not to be communicated in, or learned from, books. The skill displayed by the gynæcologist over the physician depends frequently, not so much in his superior powers as a diagnostician, as to properly applying the right quantum of alterative action, after having determined the true nature of the case. As a general rule, we do not employ a large list of these remedies—the nitrate of silver being the one upon which we most commonly rely. The frequency of its repetition, or that of any other, will depend solely upon the degree or extent of the cauterization desired or effected. The acid nitrate of mercury, potassæ cum calce and eschorotics of this class effect considerable change, and frequently destruction, more or less, of the parts brought in contact with them, and will require long intervals between the times of their application—generally from two to four weeks. We rarely use them. The milder caustics, as chromic acid, zinc, copper and nitrate of silver, should be applied more frequently ; always observing closely, however, the effects, and never re-applying so long as the effects of the preceding cauterization are visible. Our usual practice is, to produce no cauterization—*i. e.*, no eschar—but we endeavor to simply change the character of the diseased action by light applications, as frequently as the case may demand—at intervals of from three, five or more days. During these intervals, however, an appropriate treatment often overlooked or neglected, should be employed. The parts should be kept scrupulously clear by copious vaginal injections, at the temperature suited to the particular case—medicated if desired. The "uterine douche," an instrument introduced by Dr. Beiget, Physician of the Metropolitan Free Hospital, London, throwing a continuous stream of water, is the best. The common glass syringe should always be condemned—for obvious reasons—the modern gum-elastic instruments, as Davidson's, being much better, in every respect. In addition to copious injections, night and morning, if required, the patient should be instructed to introduce within the vagina appropriate medications, such as pessaries and supposotories. Supposotories may be made by taking starch and glycerine, covered by cocoa-butter—first adding any of the following articles in proper proportions: Such as Iodide of Lead, 5 grains; Tannin, 10 grains; Alum, Oxide of Zinc, or Mercurial Ointment, 20 grains; and if an anodyne is required, extract of belladonna, 3 grains. We find it good practice to apply powders, recommended by Dr. Simpson. Some pain and difficulty is frequently experienced by instrumental application of these powders. Dr. Sims uses a tampon of cotton carried to the os, by his tampon placer; we prefer, however, the instrument recommended by Prof. Thomas—a simple hard rubber tube, fitted with a piston. These applications, if employed, should be used night and morning.

We will now proceed to notice the diseased conditions which, according to our experience, are most freqently presented to the observation of the gynæcologist.

The pathological changes occurring in the uterus are not always indentical. The organ, or some part of it, may be enlarged, unattended by change of texture; and there may be alterations of texture without increase of bulk. In that condition, known as engorgement or venous hyperæmia, there is no discoverable, positive, tissue change; but there is alteration of nutrition, attended by distention of the blood-vessels, with, perhaps, slight thickening of their coats. The uterus is enlarged; the walls of the organ increased in thickness, but the consistence of the texture is unchanged. The tissues of the organ may undergo induration without inflammatory action, notwithstanding, Dr. Bennett predicates all diseased action of the uterus to inflammation. Induration is, however, most commonly the result of inflammation. Vicrow attributes induration to excessire growth of the connective tissue, while Scanzoni to increase in quantity of the muscular tissue.

Either the cervix or the body may be together or separately affected by this induration. When hypertrophy, with induration, occurs, Hewett attributes it to increase in quantity of connective tissue.

Almost all the diseases, however, coming under the observation of the gynæcologist take their initial step from inflammation occurring in the uterus, some portion of it, or in adjacent parts ; and must always constitute the chief element in their diseases—at all times demanding careful consideration and attention. A correct knowledge of the inflammatory processes occurring in the unimpregnated uterus must form the foundation of all pathological deductions in the study of uterine disorders, and a thorough acquaintance with their characteristics and progress—furnish the only guide frequently, to a clear appreciation of the nature of these diseases and the proper treatment to be employed. Inflammation may assail all the structures of the uterus, but, in a chronic form at least, it is most frequently limited to either the mucous membrane lining the cervical canal, or cavity ; or to the parenchyma of the cervix and body. It usually invades both these structures, to a greater or less extent, one, however, predominating over the other. It should be borne in mind that inflammation most generally chooses, so to speak, the cervix for the display of its diseased action. While many think otherwise, this is our experience—a majority of cases revealing inflammation of this part, and no where else. It is more generally chronic than acute. From this flow a large number of secondary affections, too often supposed to be distinct pathological changes, when only symptoms. The most common of these results are leucorrhœal discharges ; dysmenorrhœa, menorrhœgia, pruritus, prolapsus, displacements, and general debility.

In order to better understand the diseases about to be noticed, we will, for the present, consider the cervix as distinct from the body of the organ—separated from each other at the os internum. When the mucous membrane lining the canal—from the os externum to the os internum—is inflamed, it is termed *cervical endometritis ;* when the parenchyma is involved of this portion of the uterus, it is known as *cervical metritis* When it assails the mucous membrane lining the cavity, from the os internum to the fundus, it is designated *corporeal endometritis ;* and *corporeal meritis* when the parenchyma of this part is involved. This classification is not an artificial one—the diseases of the parts declaring its necessity and correctness,—by which we are enabled to better understand their nature, and is of great value in enabling us to arrive at proper methods of treatment.

As before remarked, inflammation is of most frequent occurrance in the cervix, especially when in the form of chronic cervical endometritis. Metritis, whether of the cervix or bodv, is usually induced by pathological changes following parturition or abortion. Cervical metritis is of rare occurrance in virgins, or those who have not borne children, but very common in those who have; while corporeal endometritis is of most frequent occurrance in the former class of females. When acute, all forms may speedily assail every structure of the organ, while the chronic, the usual form, commonly restricts its action to the part originally invaded.

The cervical canal, from its anatomical and physiological peculiarities, constitutes a very important study, as explanatory of the pathological conditions to which it is subjected. The canal is fusiform in shape—about one inch and a quarter in length from the os externum to the os internum. "The lining membrane of the cervix uteri—the minute anatomy of which was first thoroughly discribed by Dr. Tyler Smith—is not smooth, but furrowed, so as to present numerous depressions and elevations by which the amount of surface is very largely increased. The arrangement of two folds or plicæ varies in different cases. There are usually four prominent elevations, longitudinally placed, and four rugæ or folds of mucous membrane, and lateral transverse branches are given off from these, the whole thus acquiring a palmated aspect; and between these different elevations are seen others, more minute. The whole surface thus presents a cribriform aspect. In the recesses formed are the openings of multitudes of granular crypts." The labia of the os, however, presents "a smooth, uniform mucous surface."

*Chronic cervical endometritis* is always a distinct disease, chronic in character, from the fact that when inflammation occurs in the acute form, it spreads over the entire organ, constituting general metritis and endometritis. Chronic cervical endometritis confines its pathological ravages to the lining membrane of the cervical canal and that portion of the membrane reflected on the vaginal cervix.

When the great extent of surface comprised in its longitudinal folds, the highly vascular organization of the part, with the abundance of glandular crypts embraced within it, are remembered, we do not wonder at the frequency of its occurrence. Yet, it is a disease the study of which, with the pathological conditions attending it, as well as the proper treatment for its removal, have been the fruits of modern investigation. To Dr. J. H. Bennett, more than any one man, is the gynæcologist indebted for correct pathological principles and scientific treatment of uterine diseases. But since his publication, how rapid has been the progress of gynæcology, so happily opened up by him!

Gynæcologists—more especially M. Nonat—make two varieties of this disease. That form confined measurably to the reflected vaginal portion of the membrane, and that of the cervical canal proper. The disease may restrict itself to one or the other of these localities. The first, under a strict nosological classification, would embrace every form and variety of ulceration, which we have considered separately.

As has been stated, this is by far the most common disease of the uterine organs, according to our observation. We doubt if few women escape one or more attacks during menstrual life—we mean, of course, attacks more or less mild. It is certainly a disease, in a vast majority of cases, especially that form confined to the vaginal membrane, amenable to simple treatment—such as ablution, rest, etc. But when it becomes fixed, as it were, and distinctive inflammatory action has occurred within the cervical canal; when the glands become deeply involved and the epithelium largely destroyed, appropriate treatment applied to the part can alone eradicate the disease. In its simple form, it is only to be feared as the forerunner of other and more serious pathological changes, which often prove rebellious in the extreme. The cervix is liable to be injuriously acted upon by a variety of exciting causes : it is damaged, frequently, by coition ; lacerated during labor ; irritated by friction, both in walking and riding—especially if prolapsion has occurred. The discharge from cervical endometritis is characteristic, being poured forth by the glands under the morbid action set up. The discharge is glairy and profuse, constantly thrown out by the enlarged, elevated and dilated mouths of the follicles. Under this action the villi and papillæ become diseased and enlarged—the epithelium is finally destroyed and abrasions appear. The villi become hypertrophied, project, like soft hairs, giving the os and cervix a velvety appearance, which we have already described as granular ulceration or degeneration. This condition, however, is usually confined to the os, but it nevertheless extends up the canal. On the vaginal surface the follicles enlarge, and being ruptured, the condition occurs already noticed as follicular ulceration. Sometimes eversion of the os takes place, subjecting the protruded portion to friction and irritation upon contact with the vaginal surfaces, which intensifies and perpetuates the inflammatory condition.

It is frequently caused by coition ; to displacements, as prolapsus, retroversion, etc. ; to parturition, abortion ; to the use of pessaries, or exposure to cold during the physiological congestion of menstrual ovulation, or to clots of menstrual blood requiring expulsive efforts for their removal. It frequently occurs as a result of vaginitis, or rather, by extension of the vaginal inflammation to the cervix; also, by the constantly irritating presence of polypi, and by fissures of the cervix.

A mild form of the disease is common, and may frequently continue for weeks without attracting attention; but sooner or later, if the causes are sufficient, and the inflammation spreads, it will develop the signs of its presence. The patient will complain of fullness, weight and dragging sensations in the pelvic cavity, and will usually attribute her sensations to falling of the organ. The back and loins will ache, increased by exercise. The catamenia will be almost menorrhagic or diminished in quantity, and much pain will be had at each period. A leucorrhœal discharge will appear, thick and glutinous, and in many instances decidedly acrid and irritating to the parts over which it flows—producing inflammation and puritus of the vulva. The local symptoms are sufficiently distressing, but sooner or later the constitution will exhibit signs of impairment. According to our experienee, derangement of the digestive functions are the first to fail, and constipation occurs in almost every instance. The nervous system is, no doubt, primarily disturbed, reacting on the digestive organs. Fits of melancholy, irritability of temper and hysterical phenomena generally mark its progress; general debility will supervene, and complications will frequently occur, aggravating the original disorder—such as endometritis, cervical metritis, ovaritis, cystitis, etc.

Fortunately, however, cervical endometritis will sometimes for years limit its diseased action to its primary location. These general symptoms are not, however, positive proof of cervical endometritis. This evidence will be furnished us by physical examination. The finger being introduced, the os uteri will be felt velvety, sometimes enlarged, and, if suddenly pressed, will cause pain—in other words, the signs of granular or follicular ulceration of the os uteri will be present. Frequently, however, the finger will detect nothing abnormal, and the only indication of diseased action will be developed by raising the cervix, when pain will be felt, most commonly near the os internum. The speculum being introduced, the appearances already described under the head of glanular or follicular ulceration, will be exhibited. The os uteri, in many instances, we have observed choked by the gummy, tenacious discharge, requiring considerable time and trouble for its removal. It may frequently be drawn out to some distance, and by its elasticity spring back to its original site. Usually the cervix is normal in size—sometimes enlarged. It will generally be red, and presenting the characteristic marks of granular degeneration—the result of exfolliation of the epithelial membrane. Upon removing the mucous from the os, and cleansing the part, the cervix will be found frequently of proper size and in no manner diseased, save only the loss of epithelium. Upon further investigation by inspecting the canal, the cervix being raised and the lips separated by the hook, we will often detect ulceration within the canal. It will be necessary often to dilate the os by tents, in order to effect this exploration. Should no ulceration be found in the canal, then the lucorrhœa and other symytoms will be due to endometritis—inflammation of the mucous membrane of the uterine cavity. If the patient be a virgin, corporeal endometritis will generally be found, while if a multipora, cervical endometritis. Chronic cervical endrometritis is generally readily amenable to proper treatment. Mild forms often recover under general alterative treatment; but severer forms assume, frequently, grave complications as metritis and endometritis—the first resulting, too often, in hypertrophy, displacements and other affections destructive to health and hazardous to life.

In treating cervical endometritis, our experience justifies us in asserting that a cure turns solely upon the question of complications. If cervical endometritis exists, uncomplicated with metritis or endometritis, a large majority of such cases will be readily cured by proper treatment. This consists in hygenic measures: rest, avoidance of local excitation of the parts by sexual intercourse, and emolient and topical applications to the diseased parts. The two first are far more easily accomplished than the last.

The patient should be placed upon a non-stimulating diet. If anæmic, she should be generously nourished and given general tonic and alterative treatment. Should she have any drain upon the system, or suffer from mental depression, the first should be re. moved and the latter remedied if possible. Fresh air and gentle exercise, with proper attention to the healthful performance of the digestive functions, should form part of the treatment. Constipation should be met by appropriate measures. Mettaurer's laxitive mixture, a formula for which, is given in THE COMPANION, will, in a number of cases, accomplish this purpose, at the same time the tonic and alterative treatment should be employed. The cervix should, as far as possible, be kept clean by warm water injections; by using the gum-elastic syringe. Tincture opii, slipery elm, infusion of poppy heads, glycerine, etc., may be added to the water. If the vaginal cervix is principally diseased, the treatment already given for granular-ulceration, should be employed. The canal is found, as is often the case in our experience, to be more or less involved. Sometimes, indeed, we have found cervical endometritis to be confind to the canal. In addressing our remedies to this part, we are frequently barred enterence to their employment by the small calibre of the canal, and the tenacious mucous by which it is filled. The os is sometimes patulous, but most frequently when the vaginal cervix is not diseased the os is very little, if at all more open than normal. It will, therefore, in these cases, be necessary, first to dilate the canal with sponge-tents. We prefer the carbolized sponge-tent to any other. The sea-tangle-tent, we think, should be used when the sponge-tent is not carbolized. It is important to dilate the entire cervix. For this purpose tents should be adapted to the size of the canal. Every gynæcologist should prepare his own tents. We have no hesitancy in saying that those prepared according to the method of Mr. Robert Ellis, Obstetric Surgeon to the Chelsea and Belgrave Dispensary, England, are far preferable to any other for the purpose. Sponge-tents, it should be remembered, must be made expressly for the case in which they are to be employed. We must, therefore, determine by the case itself, the tent to be used in each particular case. Mr. Ellis selects the best sponge which is then washed in several waters to rid it of dirt, sand, etc. It is then cut in pieces about two inches in length and of such a shape as to produce, when rolled tightly over with tape, a shape somewhat fusiform"—that of the canal—" in its character. The pieces will be cut thicker or thiner according to the degree of dilitation intended." "For the larger sizes they will require to be as thick as two fingers, or even larger. For the smallest, about the thickness of the little finger. It is important to remember that the canal we propose, to dilate, by this means, is not generally larger than from one inch and a-quarter to one inch and a-half." "It will, therefore, be necessary to have some of the pieces much shorter than two inches, but very rarely, and only in abnormal elongated cases, longer than this." A square-sided instrument—a broach for instance—must be used as a stem upon which the sponge is to be compressed. "Inserting this pointed" instrument "in the centre of the piece of sponge so as to form its long axis, one of the cut edges is to be forcibly squeezed down and pressed under the opposite, which may be made to roll over it and hold it down." "In the centre of the sponge," "three or four strands of common cotton wick should be placed" before rolling, "previously dipped in pure carbolic acid." "A piece of narrow tape is then to be rolled very tightly over the apex and brought up in regular spirals, lying close to each other, to the base, when it is fastened off. The fusiform shape is to be carefully preserved in binding the tape over the dried sponge. The tent is now ready for the first coating. The best material for this," "is cocoa-butter or oil of theabroma." "In general it is useful to add a few grains of white wax—from 3 to 5—for every drachm of the oil," also, "to each ounce of the oil, one drachm and a-half of pure

(crystals, melted by heat) carbolic acid. The effect is most remarkable; the tents are withdrawn as devoid of offensive odour as when they were introduced." "The tent is dipped in the butter thus prepared, and kept in a fluid state, either over a small flame or in a hot water-bath." It should be immersed for a few seconds, so that the outer layer only of the sponge may be saturated. "The tent is then placed in a very cool place, if it has solidified, the tape is cut and unrolled. All that is now necessary to complete it is to dip it once more into the same preparation—which for this latter purpose must be much cooler than before and of about the consistency of cream. By this means a smooth and uniform covering is given the tent and it is ready for use. The tents thus prepared, must be kept in a wide-mouth, stopped bottle, so as to preserve their antiseptic properties."

(*To be continued.*)

